# LRRN4 and UPK3B Are Markers of Primary Mesothelial Cells

**DOI:** 10.1371/journal.pone.0025391

**Published:** 2011-10-03

**Authors:** Mutsumi Kanamori-Katayama, Ai Kaiho, Yuri Ishizu, Yuko Okamura-Oho, Okio Hino, Masaaki Abe, Takumi Kishimoto, Hisahiko Sekihara, Yukio Nakamura, Harukazu Suzuki, Alistair R. R. Forrest, Yoshihide Hayashizaki

**Affiliations:** 1 OMICs Science Center, RIKEN Yokohama Institute, Yokohama, Kanagawa, Japan; 2 Advanced Computational Sciences Department, RIKEN Advanced Science Institute, Wako-shi, Saitama, Japan; 3 Department of Pathology and Oncology, Juntendo University School of Medicine, Tokyo, Japan; 4 Department of Respiratory Medicine, Japan Labor Health and Welfare Organization Okayama Rosai Hospital, Okayama, Japan; 5 Japan Labour Health and Welfare organization, Kawasaki, Japan; 6 Cell Engineering Division, RIKEN BioResource Center, Tsukuba, Ibaraki, Japan; Virginia Commonwealth University, United States of America

## Abstract

**Background:**

Mesothelioma is a highly malignant tumor that is primarily caused by occupational or environmental exposure to asbestos fibers. Despite worldwide restrictions on asbestos usage, further cases are expected as diagnosis is typically 20–40 years after exposure. Once diagnosed there is a very poor prognosis with a median survival rate of 9 months. Considering this the development of early pre clinical diagnostic markers may help improve clinical outcomes.

**Methodology:**

Microarray expression arrays on mesothelium and other tissues dissected from mice were used to identify candidate mesothelial lineage markers. Candidates were further tested by qRTPCR and in-situ hybridization across a mouse tissue panel. Two candidate biomarkers with the potential for secretion, uroplakin 3B (UPK3B), and leucine rich repeat neuronal 4 (LRRN4) and one commercialized mesothelioma marker, mesothelin (MSLN) were then chosen for validation across a panel of normal human primary cells, 16 established mesothelioma cell lines, 10 lung cancer lines, and a further set of 8 unrelated cancer cell lines.

**Conclusions:**

Within the primary cell panel, LRRN4 was only detected in primary mesothelial cells, but MSLN and UPK3B were also detected in other cell types. MSLN was detected in bronchial epithelial cells and alveolar epithelial cells and UPK3B was detected in retinal pigment epithelial cells and urothelial cells. Testing the cell line panel, MSLN was detected in 15 of the 16 mesothelioma cells lines, whereas LRRN4 was only detected in 8 and UPK3B in 6. Interestingly MSLN levels appear to be upregulated in the mesothelioma lines compared to the primary mesothelial cells, while LRRN4 and UPK3B, are either lost or down-regulated. Despite the higher fraction of mesothelioma lines positive for MSLN, it was also detected at high levels in 2 lung cancer lines and 3 other unrelated cancer lines derived from papillotubular adenocarcinoma, signet ring carcinoma and transitional cell carcinoma.

## Introduction

Mesothelioma is one of the most fatal and difficult to treat cancers in the world and in Japan the median survival rate from diagnosis is 9 months with 94% of patients not surviving at 3 years [1]. In recent years the incidence of diagnosed cases of mesothelioma in Japan has doubled from 500 cases in 1995 to 1068 in 2007. The majority of mesothelioma cases can be directly linked to asbestos exposure and is estimated to be responsible for at least 90% of all cases [2]. Asbestos has long been used for its insulating properties and was used for insulation around pipes, inside ceiling cavities, brake linings and compounded into asbestos composite sheeting for an inexpensive heat and fire resistant building material. In the 1960s it was recognized that occupational asbestos exposure was a strong risk factor for the development of mesothelioma [3], [4], [5]. At particular risk were asbestos mine workers at locations such as Wittenoom Western Australia [6] and Western Cape South Africa [5]. The realization that Asbestos exposure was responsible led to legislation in multiple countries which progressively reduced the use of asbestos and potential exposure and finally led to complete bans in 52 countries [7]. Despite this, the past large scale use of asbestos means that there is still a large amount of it in the general community that needs to be safely removed, with demolition workers likely to be at risk. The latent period between exposure and diagnosis is in the order of 20–40 years meaning that workers who were exposed a long time ago may still develop the disease [8]. Considering these two factors, the number of mesothelioma cases is likely to continue to rise at least for the next 20 years.

For definitive diagnosis, immunohistochemical staining of biopsy material for epithelial cell adhesion molecule (EPCAM) and Vimentin (VIM) have been used to discriminate mesothelioma from other lung cancers [9], [10], however more recently a microRNA based test with a higher accuracy has been developed and made available to doctors [11]. These are important for determining treatment however none of these are used for early detection. Recently a kit (MESOMARK [12], [13]) for detection of soluble mesothelin related peptides in patients' blood has made it to market. This ELISA based kit is currently the most specific and sensitive to market [14]. Mesothelin was originally identified as megakaryocyte potentiating factor (MPF), and later termed mesothelin when it was shown that an antibody against it reacted with ovarian cancers and malignant melanomas [15], [16] and as early as 1999 it was shown that mesothelin related peptides could be detected in the serum of ovarian cancer patients [17]. Mesothelin is produced as a transmembrane protein which is then cleaved to release (MPF). Considering the poor prognosis and the long latent period before diagnosis there is both a need for earlier diagnosis and a real opportunity to develop earlier tests (10–20 years window). In general earlier diagnosis means improved likelihood of survival [18].

With this in mind we set out to identify additional mesothelial lineage markers using a comparative functional genomics approach. Gene expression profiling of dissected mouse mesothelium and other tissues was used to identify candidate biomarkers enriched in mesothelium. We then went on to filter these lists by first focusing on candidate biomarkers that were likely to be secreted or transmembrane proteins and then validating the specificity of the markers by quantitative in-situ hybridization in mouse tissues and real time PCR (qRTPCR) across a panel of human cancer cell lines and normal lung cells and mesothelial cell RNAs.

## Materials and Methods

### Ethics Statement

All animal work was carried out according to institutional and national ethical guidelines, and approved by the Yokohama Animal Experiment Review Committee, Plan19-002. Similarly all human work was carried out according to institutional and national ethical guidelines. The human mesothelioma sample used in the immunohistochemistry was made available anonymously from the autopsy material collection at the Department of Pathology and Oncology, Juntendo University School of Medicine. The human primary mesothelial cells were purchased from Zenbio. The human primary cells were purchased from Zenbio, Sciencell, Cell Applications and Lonza.

### Animal and Tissue sources

The tissues of 7-week-old male mice on C57BL/6 backgrounds were used for RNA preparation and for ISH. For RNA preparation, mice were anesthetized, then sacrified, then their organs were removed or harvested and immediately washed with Phosphate-Buffered Saline (Invitrogen) and transferred into RNA later (Ambion). Two sets of mesothelial tissue were prepared from 200 mice. Peritoneal mesothelium was prepared as one source and pleural and pericardial mesothelium were pooled as the second source. Lung, liver, brain, heart, skeletal muscle, subcutaneous adipose, testis, bladder and kidney tissues were also dissected as required for RNA extraction.

For ISH, Mice were anesthetized with diethyl ether and transcardially perfused with PFA solution (4% paraformaldehyde, 4% sucrose in sodium phosphate buffer pH 7.5) and then with Bouin's solution (71% saturated picric acid [v/v], 24% formalin [v/v], and 5% acetic acid [v/v], adjusted to pH 4.0 with sodium hydroxide). The brains were then removed, fixed with original Bouin's solution for 3 days, and embedded in paraffin.

All procedures involving animals and their care were carried out in accordance with the directives of RIKEN's Institutional Animal Care and Use Committee.

### RNA extraction

Total RNA was purified using the RNeasy mini kit (Qiagen) and RNeasy Lipid Tissue Midi kit (Qiagen) according to manufacturer's instructions. The quality of the extracted RNA was confirmed with Agilent 2100 Bioanalyzer.

### Microarray

Five hundred nanogram of total RNA was amplified using the Illumina TotalPrep RNA Amprification Kit (Ambion), according to manufacturer's instructions. cRNA was hybridized to Illumina Mouse Sentrix-6 Expression BeadChip Ver.1, according to standard Illumina protocols. Chips scans were processed using Illumina BeadScan and BeadStudio software packages and summarized data was generated in BeadStudio (version 3.1). Data was quantile normalized using Lumi and B-statistics were calculated using Limma (both R packages in Bioconductor) [19], [20]. Meosthelial tissue gene expression was compared pairwise against each other tissue, and only genes where the B-statistic was greater than 0 for all cases and the average expression in the mesothelial tissue was greater than 2 fold for all other tissues was the expression considered mesotheial specific. All microarray data is MIAME compliant and the raw data has been deposited in a MIAME compliant database (GEO (ID pending)).

### qRTPCR

qRTPCR was carried out as described previously [21]. Primers used are listed in **Table S1**. Primers for human MSLN were from Jirsova et al [22], all other primers were designed in house.

### In situ hybridization (ISH)

ISH was carried out using PCR generated probes with FANTOM1 and FANTOM2 cDNA clones or genomic DNA as templates (**Table S2**). ISH was carried out as described previously [23].

### Human total RNA samples

Primary cells or RNA were purchased: Mesothelial cells– (Zenbio, Cat: F-MES-F), small airway epithelial cells (Lonza, Cat: CC-2547), lung fibroblasts (Cell applications, Cat: CA506-R10f), alveolar epithelial cells (Sciencell, Cat: SC3205), bronchial epithelial cells (Sciencell, Cat: SC3215), bronchial smooth muscle cells (Sciencell, Cat: SC3405), tracheal smooth muscle cells (Sciencell, Cat: SC3415), retinal pigment epithelial cells (Sciencell, Cat: SC6545) and urothelial cells (Sciencell, Cat: SC4325). The cell lines (ACC-MESO-1, ACC-MESO-4 [24], LC-1F, EBC-1, LU65, Lu99, PC-14, A549, Lu-134-A, LK-2, IA-5, T3M-10, HeLa, OS-RC-2, JMSU1, TGBC2TKB, TGBC18TKB, A431, Kato III and WERI-Rb-1) were provided by the RIKEN BRC through the National Bio-Resource Project of the MEXT, Japan. The additional mesothelioma cell lines were purchased from the American type culture collection (NCI-H28, NCI-H226, NCI-H2052, NCI-H2452 [25]) and the European Collection of Cell Cultures (Mero-14, Mero-25, Mero-41 [26], Mero-48a, Mero-82, Mero-83, Mero-84, Mero-95 [27], No36, ONE58 [28]). Total RNA was prepared using QIAGEN miRNeasy columns (Cat: 217004) according to the manufacturer's instructions. Adult lung RNA was purchased from Ambion (Cat: AM6000).

### Immunostaining

Antibodies against synthetic peptides from UPK3B, NKAIN4 and LRRN4 were ordered from IBM as a custom service. Immunostaining was carried out as described previously [29], [30].

## Results

### Identification of mesothelial markers in mouse

Two sources of mesothelial tissue were dissected from 7 week old C57/BL6 mice. Total RNA was extracted from peritoneal mesothelium and a pool of pleural and pericardial mesothelium. Triplicate gene expression analysis was carried out using illumina microarrays on the mesothelium samples and unrelated tissue RNAs (adipose, bladder, heart, liver, lung, skeletal muscle). Data was normalized using the Lumi [19] package of bioconductor and differential gene expression between the mesothelium and other tissues was determined using B-statistics in the Limma [20] package. Peritoneal mesothelium or the pleural/pericardial pool was compared against each of the tissues separately. Only probes that showed significance (B-statistic >0 and at least 2 fold higher expression) in all comparisons (ie mesothelium vs heart, mesothelium vs liver etc.) were considered putative mesothelium specific markers. 460 probes (corresponding to 311 loci) passed this test as being over-expressed in at least one of the mesothelium sources compared to all the other tissues (**Table 1**, **Table S3**). Surprisingly only 48 of these were significant for both mesothelium sources. 223 were only over-expressed in the peritoneal mesothelium samples and 45 were only over-expressed in the pleural/pericardial mesothelium samples. Of the 48 genes whose expression was enriched in both mesothelial sources seven were previously associated with the mesothelial lineage or mesothelioma (Aldh1a2 [31], Crip1 [32], Has1 [33], Prrx2 [34], Upk3b [35], Wnt2b [36], and Wt1 [37]).

**Table 1:** Mesothelium enriched genes identified by microarray analysis.

	Mesothelium enriched genes
Both sources	2010001M06Rik , 2010107G12Rik , 4930415N12Rik , 4930455G09Rik , A730017C20Rik , A730046J19Rik , Acot11 , **Aldh1a2** , Anxa9 , Aplp1 , Ccdc109b , Cdon , Celf4 , Chst4 , Cldn15 , **Crip1** , Cybrd1 , D930030F02Rik , Dcaf12l2 , Fam70a , Flrt2 , Gpm6a , **Has1** , Ildr2 , Isyna1 , Lgals7 , Lrrn4 , Nkain4 , Nxnl2 , Pkhd1l1 , **Prrx2** , Rpp25 , Rspo1 , Saa3 , Serpinb2 , Sez6l , Slitrk4 , Smpd3 , Spns3 , Sulf1 , Tmem114 , Tnfrsf11b , Trem2 , Tubb2b , **Upk3b** , Vit , **Wnt2b** , **Wt1**
Peritoneal only	1110012D08Rik , 1110064A23Rik , 1500015O10Rik , 1700023F06Rik , 2410039M03Rik , 2600006K01Rik , 2900073C17Rik , 4930533K18Rik , 5430435G22Rik , 5730409K12Rik , 6330406I15Rik , 7330423F06Rik , 9530077C05Rik , Abcg4 , Accn1 , Adamts19 , Adamts2 , Aebp1 , Agap1 , AI661453 , AI747448 , Ak5 , Alox15 , Anks6 , Anln , Arhgap29 , AY761184 , AY761185 , B3gnt9-ps , Bicc1 , C2 , C330020E22Rik , Cacna1g , Capn6 , Casp12 , Ccdc80 , Ccin , Ccrl1 , Cd109 , Cd34 , Cdh11 , Cdh3 , Cercam , Cgnl1 , Chrd , Clca6 , Clec11a , Clec4d , Clps , Col6a1 , Col6a2 , Col6a5 , Colec12 , Cox6b2 , Cplx2 , Cpt1c , Cpz , Crb2 , Creb3l1 , Csrp2 , Dbn1 , Dcn , Ddr1 , Defa17 , Defa20 , Defa22 , Defa23 , Defa3 , Defa5 , Defa6 , Defa-rs1 , Dgkb , Dhrs9 , Disp1 , Dlc1 , Dlk1 , Dmrt3 , Doc2b , E330013P04Rik , Ecm1 , Efemp1 , Emilin2 , Eno2 , Enox1 , Enpp2 , Ephb6 , Espn , Etv1 , Fam114a1 , Fam180a , Fam184a , Fbln2 , Fbn1 , Fez1 , Fgf1 , Fgf18 , Fgf5 , Fgf9 , Fkbp10 , Fkbp14 , Fkbp9 , Flrt1 , Fosb , Gabrb1 , Gabrq , Galns , Galntl2 , Garnl3 , Gas6 , Ghrh , Gjb3 , Glb1l , Glt8d2 , Gm12680 , Gng4 , Gpr85 , Gria3 , Guca1a , Gxylt2 , Hif3a , Htra1 , Ifitm1 , Igfbp6 , Ikbip , Il31ra , Il7 , Ildr2 , Isl1 , Islr , Itln1 , Kcnab1 , Kcnip3 , Kirrel , Krt76 , Lamc3 , Leprel2 , Lgals1 , Lgals2 , Loxl1 , Loxl2 , Lrpap1 , Ltbp3 , Man2b2 , Mapk4 , Mdk , Mfap5 , Miat , Mmp16 , Mmp23 , Mmp24 , **Msln** , Mtmr11 , Muc13 , Muc16 , Muc2 , Nbl1 , Ndrg1 , Neo1 , Nfkbiz , Nid2 , Ninl , Nkx2-3 , Nlgn1 , Nrxn2 , Nxph1 , Oasl2 , Olfml2b , Olfr1036 , Pcolce , Penk , Piwil2 , Plat , Plekha4 , Prg4 , Ptgis , Rassf2 , Rcn3 , Reg3b , Rnf112 , Rtn1 , Scg5 , Scrn1 , Selp , Selv , Sema5a , Serpinh1 , Sertad4 , Sfxn3 , Sgsm2 , Slc1a7 , Slc23a3 , Smpd3 , Smpdl3b , Snord123 , Sorcs1 , Sox8 , Spink3 , Ssbp3 , Stag3 , Steap2 , Stra6 , Sulf1 , Sv2a , Svs5 , Tmem119 , Tmem151a , Tmem98 , Tnxb , Trim9 , Tro , Ttll12 , Ttyh1 , Tulp2 , **Upk3b** , Vasn , Vat1l , Vmn2r26 , Wasf1 , Wnt6 , Zbtb7c , Zbtb8a , Zfp185 , Zg16
Pleural + pericardial only	1190002F15Rik , 2810417H13Rik , A530032D15Rik , A530050N04Rik , AB124611 , AI324046 , Aurkb , B230209E15Rik , Bfsp2 , Btg3 , Calcb , Csprs , Cxcl13 , Cybrd1 , Dntt , Expi , Fcna , Gng13 , Gpr176 , Hist1h2an , Igfbp5 , Kif22 , Oosp1 , Plac1l , Prss16 , Rag1 , Rilpl2 , Sdc3 , Sema3d , Serpina3b , Slc2a6 , Spo11 , Stac2 , Tbata , Tm4sf5 , Tnfrsf17 , Uhrf1 , Xpnpep2 , Zbtb32

Note: Gene names in bold have been previously associated with mesothelioma or the mesothelial lineage.

Interestingly Msln a previously reported marker of the mesothelial lineage was only found to be significantly more highly expressed in the peritoneal mesothelium and not in the pleural and pericardial sample. For the genes enriched in only one type of mesothelium we found separate classes of genes involved in immune response. In the peritoneal only list, CD34 was 4 fold higher expressed than in other tissues, and eight of the known defensins were also enriched (11–200 times that of any other tissue). However in the pleural + pericardial mesothelium there was enrichment for immunoglobulin heavy and light chains (2.3–76 times that of any other tissue) suggesting possible B or plasma cell contamination. This suggests different sets of hematopoietic cells are associating with the mesothelium from different locations.

To further investigate the gene sets we carried out gene ontology analysis using the GOstat tool [38]. There was no gene ontology enrichment for the genes highly expressed in the pleural/pericardial mesothelium, but the list of genes expressed in both mesothelial tissues and the peritoneal only samples were enriched for genes involved in cell adhesion and extracellular space. The peritoneal set was also enriched for genes involved in developmental processes and multicellularity (**Table S4**).

### qRTPCR testing of candidate markers in selected mouse tissues

To further focus on biomarkers of mesothelium we chose a set of 36 candidates for further confirmation by qRTPCR which were likely to be secreted, transmembrane proteins or novel. This included 11 of the genes enriched in both mesothelium sources, 20 genes only enriched in peritoneal mesothelium and 5 genes only enriched in the pleural and pericardial mesothelium (**Table S1**).

The mesothelium specific expression of the 36 candidate biomarkers was then tested by qRTPCR across a broader selection of tissue RNAs (pleural + pericardial mesothelium, peritoneal mesothelium, lung, adipose, heart, liver, brain, kidney, thymus, spleen, intestine, testis) and 14 candidates that were still shown to be meosthelial specific were further tested against an additional 5 other tissues (bladder, skeletal muscle, colon, uterus, ovary) and also against mesothelium from female mice to confirm there were no sex specific differences. This secondary screening reduced the number of candidates to 13, (including 4 of the original identified in both tissues and 9 identified in peritoneal only). The top 10 most mesothelial specific of these were then chosen for further screening by in-situ hybridization.

### In-situ hybridization of 10 candidate biomarkers

Of the 10 candidate biomarkers identified in the study, several have previously been associated with the mesothelial lineage or mesothelioma, these include the prototype mesothelial lineage marker mesothelin (Msln)[15] and others such as uroplakin 3B(Upk3b) [35], prostaglandin I2 (prostacyclin) synthase (Ptgis) [39] and insulin-like growth factor binding protein 6 (Igfbp6) [40]. The remaining six, leucine rich repeat neuronal 4(Lrrn4), Na+/K+ transporting ATPase interacting 4(Nkain4), proteoglycan 4(Prg4), matrix metallopeptidase 23(Mmp23), family with sequence similarity 180, member A (Fam180a), selectin P(Selp) have not been previously associated with the mesothelial lineage. Although Prg4 specifically has not been previously associated with the mesothelial lineage, these cells are known to produce proteoglycans to facilitate lubrication within the peritoneum [41].

**Figure 1:**
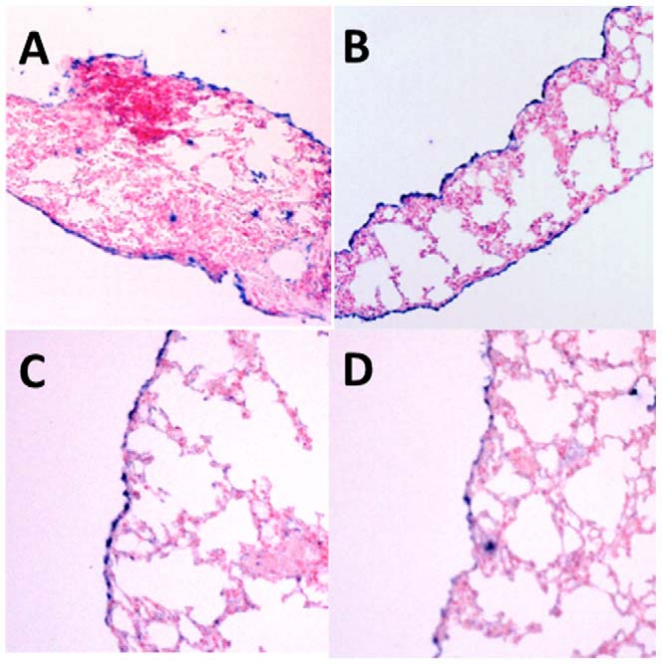
In-situ hybridization images of mouse lung tissue with pleural mesothelium intact stained for A) mesothelin, B) uroplakin 3b, C) leucine rich repeat neuronal 4 and D) Na+/K+ transporting ATPase interacting 4. Note the mesothelial staining in blue.

In-situ hybridization was carried out across a selection of mouse tissues including lung and heart (both with mesothelium intact), liver, kidney, spleen, intestine, testis and thymus. For the previously reported markers Msln, Upk3b, Ptgis and Igfbp6 mesothelial staining was observed. In addition two of the novel markers, Nkain4 and Lrrn4 showed mesothelial staining (**Figure 1**, **Figure S1**).

### LRRN4 is a novel human mesothelial lineage marker

To confirm the specificity of these markers in human mesothelium and mesothelioma we designed primers against human MSLN, UPK3B, NKAIN4 and LRRN4 (**Table S1**) and tested their specificity by qRTPCR against a panel of 45 human total RNAs. The panel was selected to contain normal lung tissue, primary cells of the lung including a range of epithelial cells, smooth muscle cells and fibroblasts, primary mesothelial cells from 2 donors, 16 mesothelioma cell lines, 10 lung cancer cell lines and 8 unrelated cancer cell lines. Primary retinal pigment epithelial cells and urothelial cells were also included as unrelated epithelial cells.

**Figure 2:**
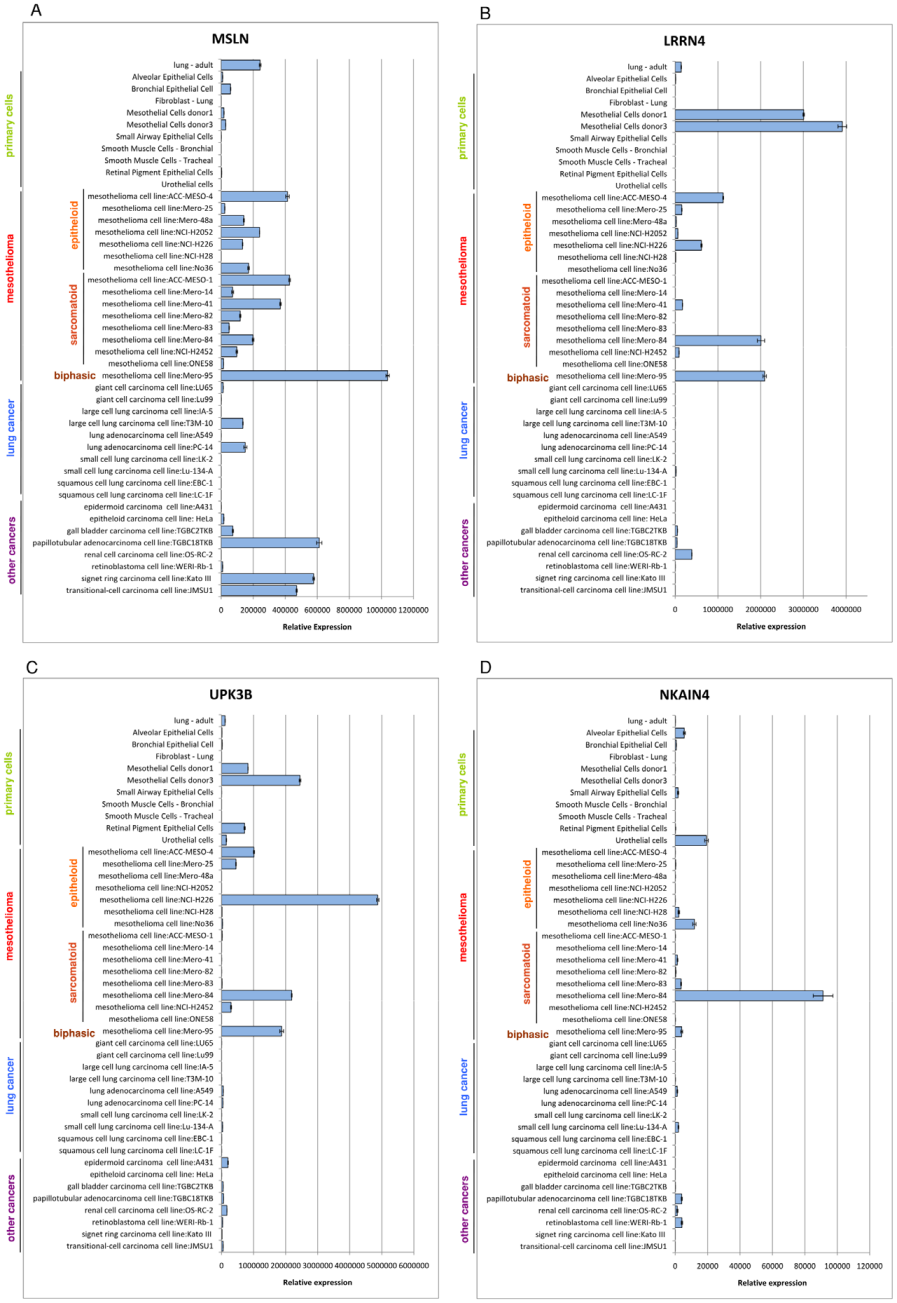
Expression of A) MSLN, B) LRRN4, C) UPK3B, D) NKAIN4 across a panel of primary human cell types and cancer cell lines measured by qRTPCR.

First considering the primary cells, MSLN, LRRN4 and UPK3B were detected in the primary mesothelial cells, however LRRN4 showed the greatest specificity and was only detected in primary mesothelial cells. MSLN and UPK3B however were less specific and were detected in bronchial epithelial cells and alveolar epithelial cells or retinal pigment epithelial cells and urothelial cells, respectively (**Fig. 2**).

Despite this, testing the cell line panel revealed that MSLN still appears to be a better marker of mesothelioma than LRRN4 and UPK3B. MSLN was detected in 15 of the 16 mesothelioma lines tested and for 13 of these the expression level was higher than that of the primary mesothelial cells. In the case of LRRN4 and UPK3B the expression was only detected in 8 or 6 of the tested lines suggesting these markers are down-regulated or lost in cancer.

Despite the higher fraction of mesothelioma lines positive for MSLN, it does not appear to be a completely mesothelioma specific marker as it was strongly expressed in the large cell lung carcinoma cell line T3M-10 and moderately expressed in the lung adenocarcinoma line PC-14. Surprisingly MSLN was also detected at high levels in the duodenal papillotubular adenocarcinoma cell line TGBC18TKB and at moderate levels in the gastric signet ring carcinoma line Kato III, bladder transitional cell carcinoma line JMSU1, gall bladder carcinoma line TGBC2TKB and even weakly in the ovarian epitheloid line HeLa (**Fig. 2**).

Finally at this stage of the screening NKAIN4 failed to validate as a marker of human mesothelium or mesothelioma, it was most highly expressed in primary urothelial cells and amongst the lung associated cell types it was much more highly expressed in alveolar and small airway epithelial cells than mesothelial cells (**Fig. 2**).

### Immunostaining of mesothelial biomarkers in human mesothelioma

**Figure 3:**
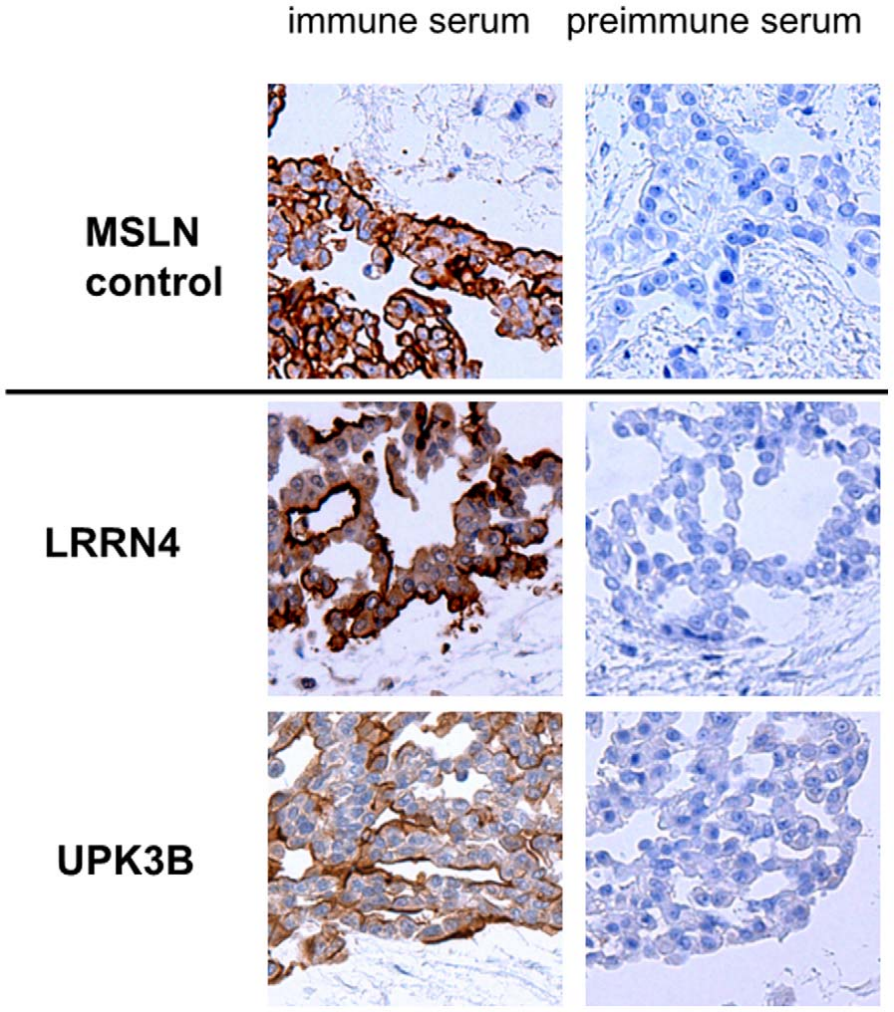
Immunohistochemisty of a human epithelial mesothelioma stained with antibodies against MSLN, LRRN4, UPK3B.

With the aim of developing these biomarkers for diagnostic testing in the future we set out to generate antibodies against UPK3B, and LRRN4. Three synthetic peptides were generated for each of the three proteins and antibodies raised in Rabbits (**Figure S2**). The best antibody for each protein was then chosen for further testing. Immunohistochemistry staining of a human epithelial mesothelioma sample was then carried out using these antibodies and a positive control against MSLN [29], [30]. Mesothelial specific signal was observed for all three antibodies, with a pattern similar to that of the MSLN positive control (**Fig 3**). Cell surface staining was observed for MSLN, LRRN4 and UPK3B.

## Discussion

We have described a comparative functional genomics screen to identify novel markers of the mesothelial lineage with potential for secretion. From this screen we identified several putative biomarkers and went on to generate antibodies against LRRN4, and UPK3B. In terms of specificity, LRRN4 appears to be the most specific marker of the mesothelial lineage, however when tested across a panel of 16 mesothelioma cell lines is only detected in 8, and down-regulated in the remaining 8 suggesting it may be lost during tumor progression (or adaptation to cell culture). We show that Mesothelin is detected in 15 of 16 cell lines and is upregulated relative to primary mesothelial cells in 13.

Interestingly qRTPCR across a panel of human RNAs demonstrated that MSLN is not restricted to mesothelium or mesothelioma. In normal primary lung associated cells MLSN is detected at a higher level in bronchial epithelial cells than mesothelial cells. In tumor cell lines, expression was detected in mesothelioma cell lines, but was also expressed at similar levels in a large cell lung carcinoma, duodenal papillotubular adenocarcinoma, signet ring carcinoma, transitional cell carcinoma and gall bladder carcinoma. Weak expression in HeLa cells was also observed, which is consistent with a list of biomarkers reported as upregulated in cervical cancer [42]. MSLN is also upregulated in other cancers including ovarian cancer, and pancreatic ductal adenocarcinoma [43] and in its original description, soluble mesothelin was found to be a serum marker for ovarian cancer [17]. This has implications for the MESOMARK [12], [13] kit for detection of soluble mesothelin as other tumor types including some lung cancers with similar presentations may produce soluble mesothelin.

Regarding the other two markers. UPK3B has been reported previously as a marker of the mesothelial lineage [35]. However as its name suggests it is also expressed in urothelial cells and we also find strong expression in retinal pigment epithelial cells. LRRN4 however has not been previously associated with mesothelioma or the mesothelial lineage. LRRN4 (also known as NLRR4 and C20orf75) is a leucine rich repeat protein that was originally identified as a neuronal protein involved in long term memory formation [44], interestingly in this publication they note strong expression in the lung and ovary, similar to the pattern for mesothelin. Although LRRN4 has not been associated with mesothelioma it has been shown previously to be regulated in response to acute lung injury [45] and in Chronic Obstructive Pulmonary Disease (COPD) [46] suggesting it may play a role in response to lung damage. It is also upregulated in the adipose of Mif knockout mice [47]. MIF (macrophage migration inhibitory factor) is implicated in COPD [48] and is a cytokine involved in maintenance of proinflammatory response in macrophages [49]. It is hypothesized that there is a link between asbestos exposure, chronic inflammation and mesothelioma [50] however whether there is any significance in this link is unclear.

The observation that LRRN4 is highly expressed in the primary mesothelial cells, is lost in 8 of the mesothelioma cell lines and is expressed at lower levels in the remaining 8 lines is of interest. There appears to be no correlation between mesothelioma subtypes (our collection included 7 epitheloid, 8 sarcomatoid and 1 biphasic type cell line). Together these data paint a picture of MSLN as a less lineage specific marker that is upregulated in cancer to promote proliferation and migration of tumor cells [51], while LRRN4 and UPK3B are more specific lineage markers that provide no competitive advantage to the tumor so can be lost during tumor progression. With this in mind it may be of interest to consider whether loss of LRRN4 or UPK3B expression correlates with later stage and poorer prognosis, but is beyond the scope of the current manuscript.

In conclusion, we identified 311 putative markers of the mesothelial lineage including 48 that were found in pleural, pericardial and peritoneal mesothelium (it is yet to be shown whether they are also expressed in the other rare mesothelial populations at the tunica vaginalis and tunica serosa uteri). We go on to demonstrate that polyclonal antibodies against two of these, LRRN4 and UPK3B react with human mesothelioma tissue localised to the plasma membrane. With this in mind we are currently exploring the development of LRRN4 ELISA based assays for detection of mesothelioma antigens in patient body fluids.

## Supporting Information

Figure S1 Extended In-situ hybridization panels for 10 candidate biomarkers and GAPDH control. Shows staining pattern across a panel of tissues (Lung, Heart, Liver, Kidney, Spleen, Intestine, Testis and Thymus).
(PDF)

Figure S2 Peptide sequences used to raise antibodies for LRRN4 and UPK3B.
(PDF)

Table S1 qRTPCR validation. Includes Illumina probeID, Gene symbol, primer sequences and CT values across a panel of tissues.
(XLS)

Table S2 In-situ hybridization probes. Includes Symbol, target and primer sequences.
(XLS)

Table S3 Genes enriched in mesothelium, including B-statistic and fold change.
(XLS)

Table S4 Gene ontology enrichment analysis of mesothelial enriched gene sets.
(XLS)
