# Supplementary material for: LRRN4 and UPK3B Are Markers of Primary Mesothelial Cells
Source: PLoS One. 2011 Oct 3;6(10):e25391. doi: 10.1371/journal.pone.0025391 (PMC3184985; doi:10.1371/journal.pone.0025391)

Supplementary Figure 1

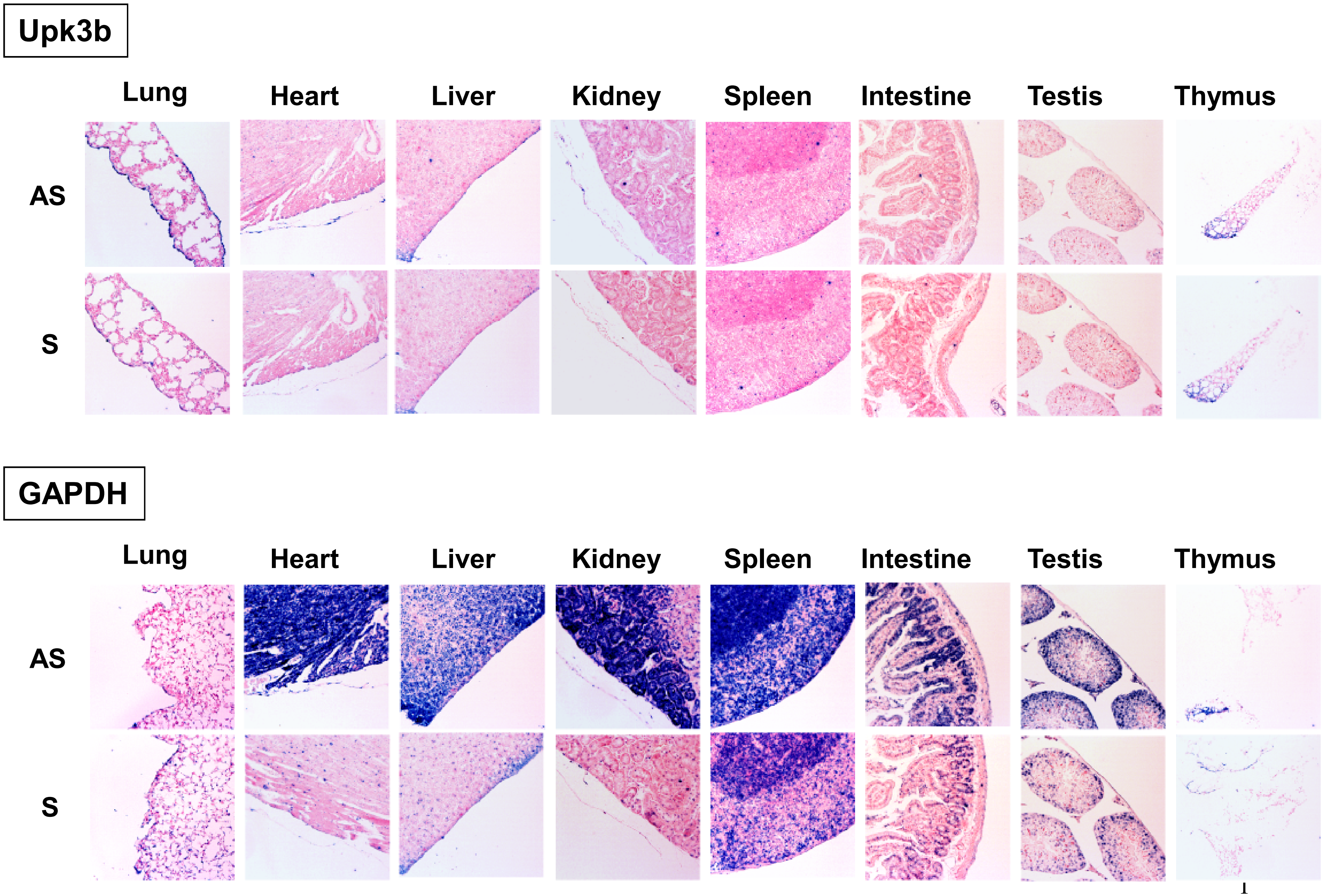

Supplementary Figure 1 (continued)

C030019F02Rik, B430119L13Rik

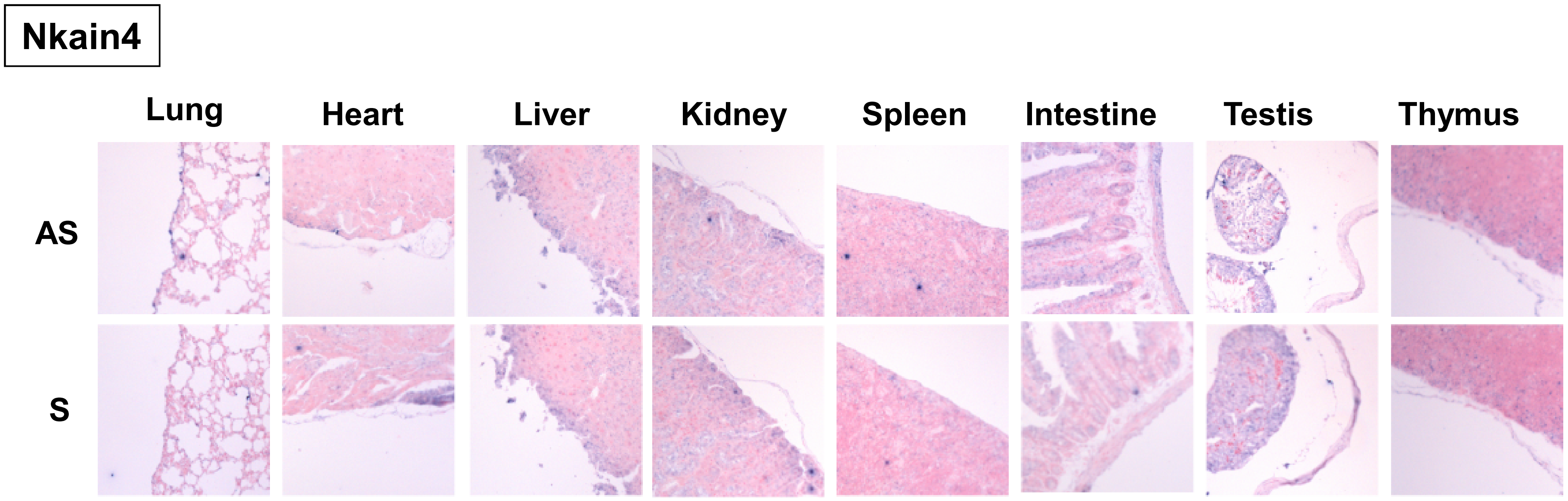

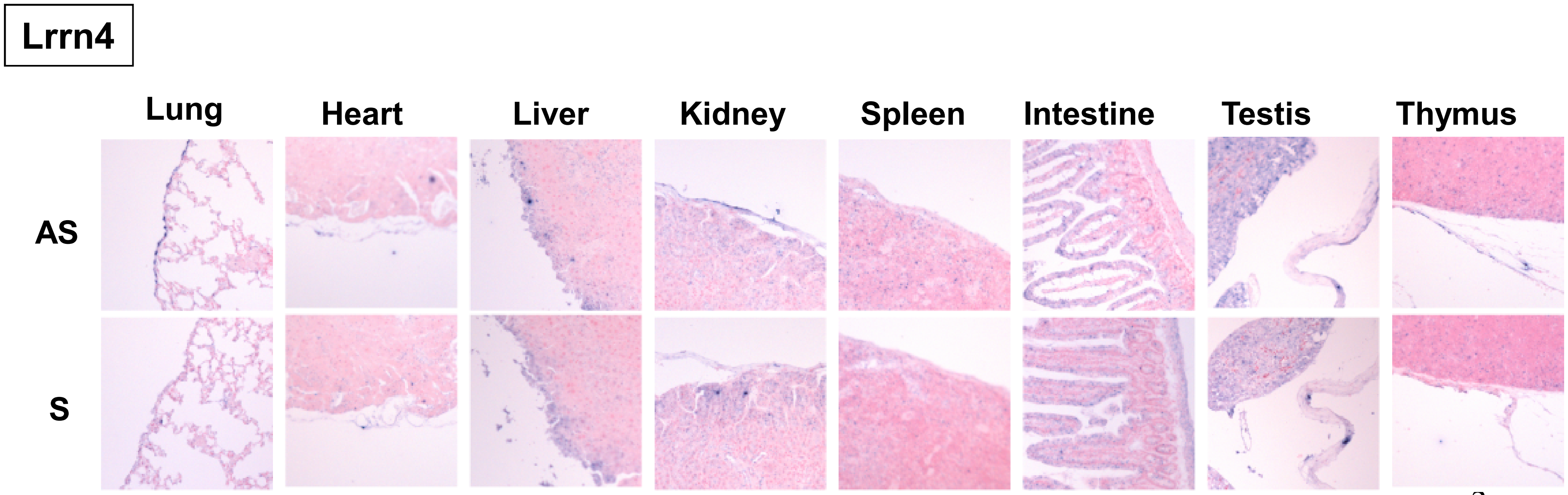

Supplementary Figure 1 (continued)

Msln

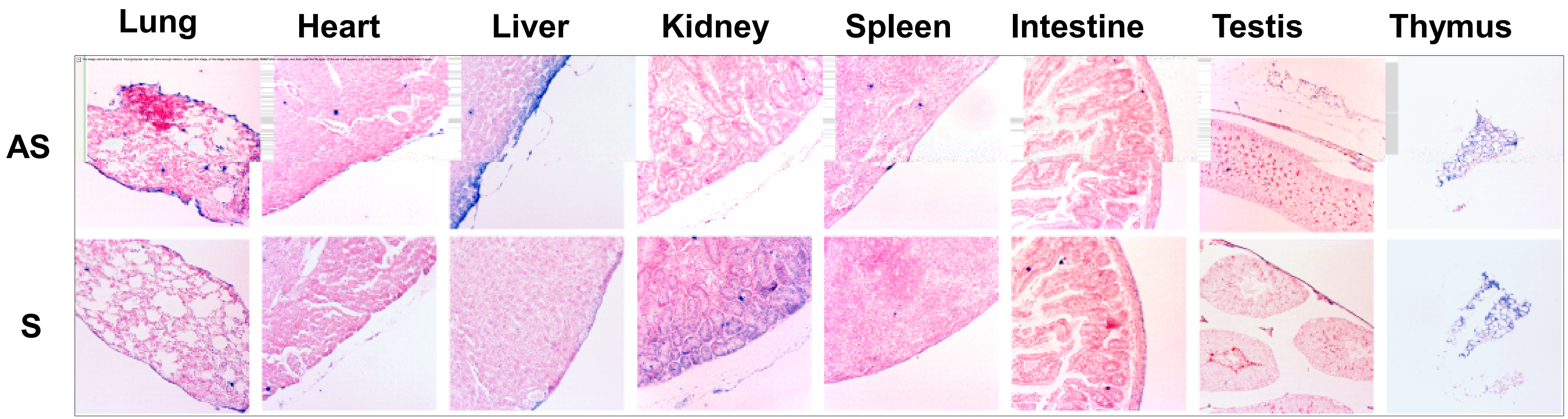

Ptgis

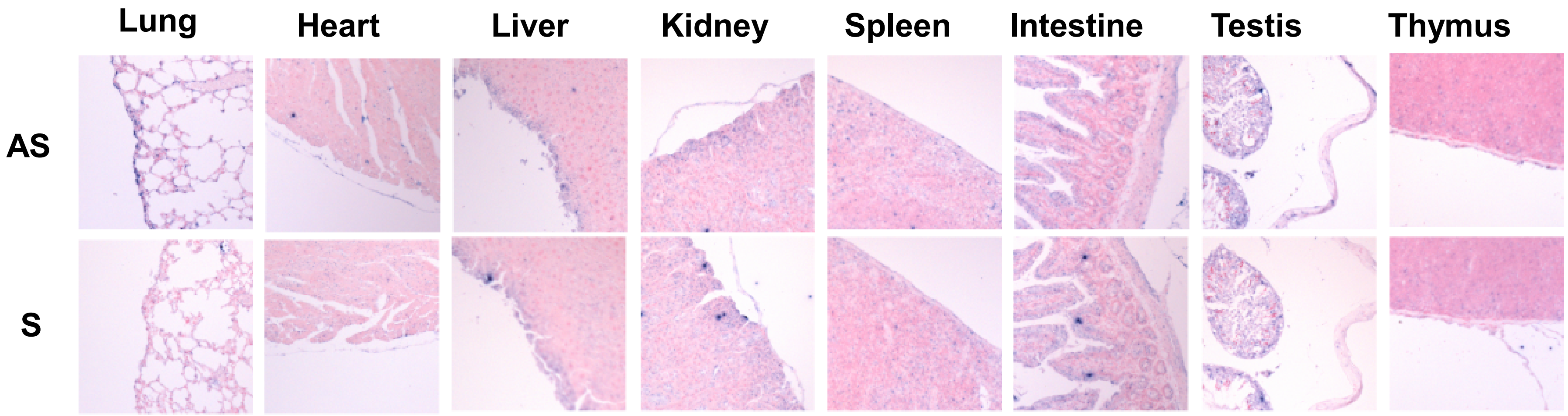

Supplementary Figure 1 (continued)

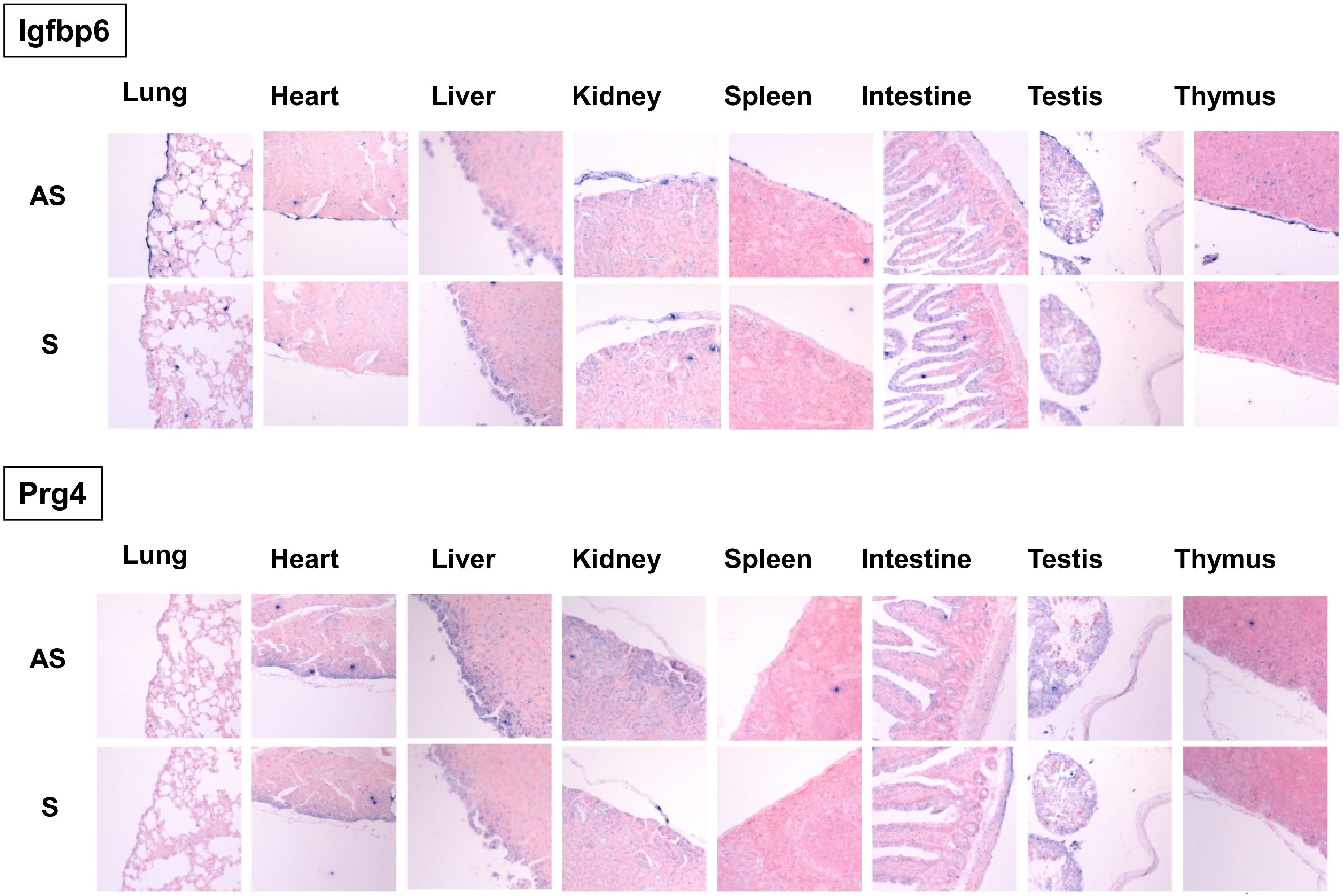

Supplementary Figure 1 (continued)

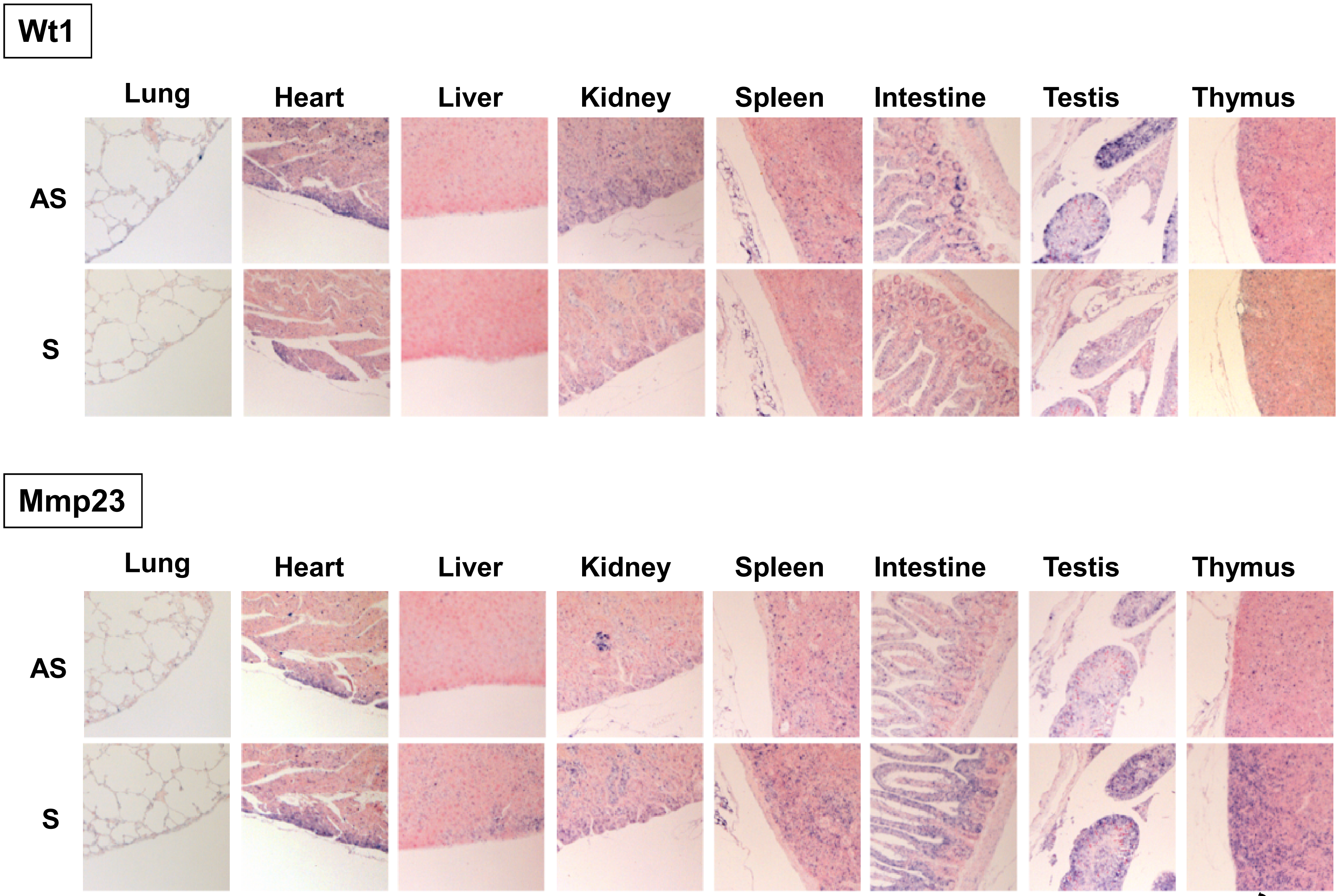

Supplementary Figure 1 (continued)

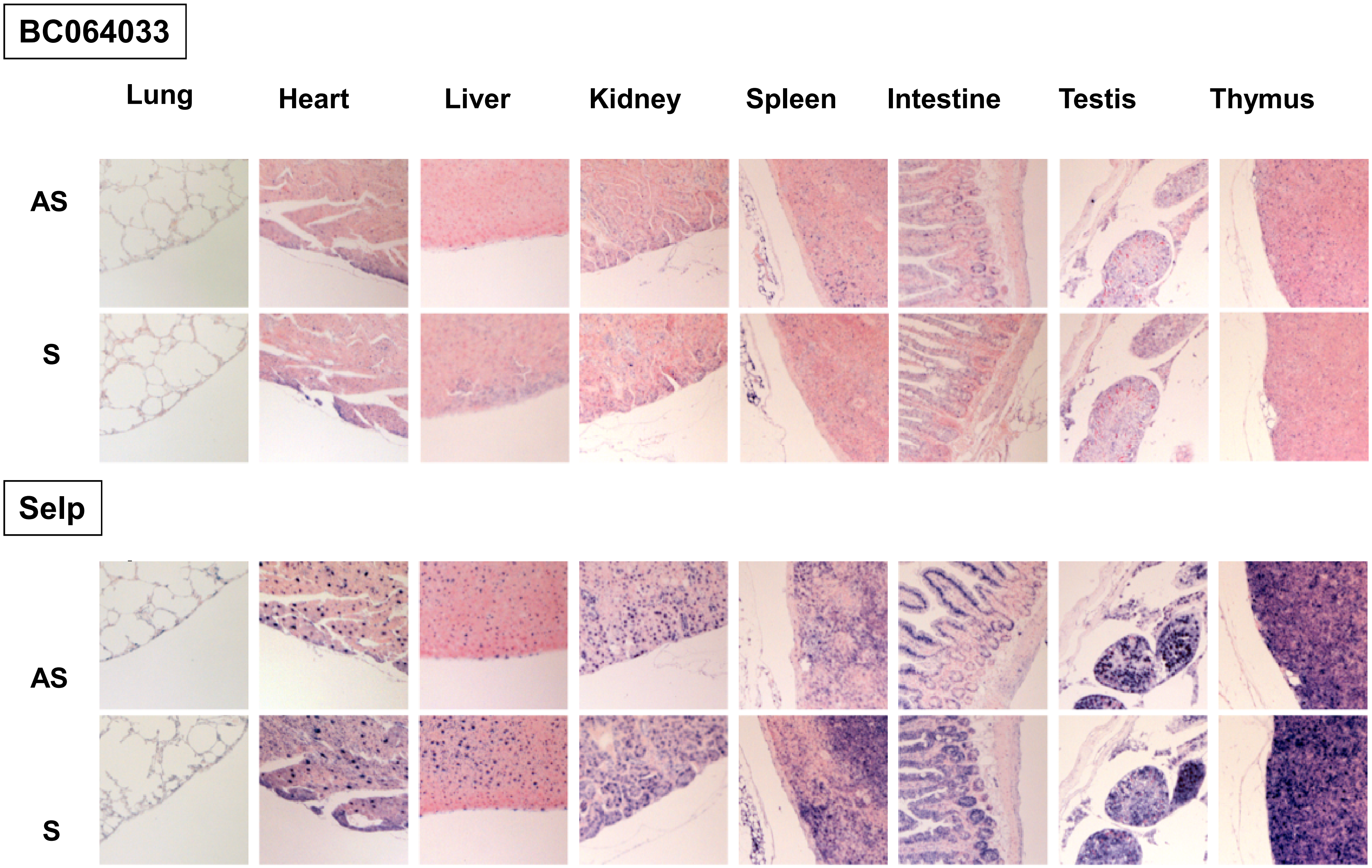

Supplement: Figure S1 — Extended In-situ hybridization panels for 10 candidate biomarkers and GAPDH control. Shows staining pattern across a panel of tissues (Lung, Heart, Liver, Kidney, Spleen, Intestine, Testis and Thymus). (PDF) [file pone.0025391.s001.pdf]
