# Supplementary material for: LRRN4 and UPK3B Are Markers of Primary Mesothelial Cells
Source: PLoS One. 2011 Oct 3;6(10):e25391. doi: 10.1371/journal.pone.0025391 (PMC3184985; doi:10.1371/journal.pone.0025391)
Supplement: Figure S2 — Peptide sequences used to raise antibodies for LRRN4 and UPK3B. (PDF) [file pone.0025391.s002.pdf]

Supplementary Figure 2

LRRN4(NM_152611)

MRQTLPLLLL TVLRPSWADP PQEKVPLFRV TQQGPWGSSG SNATDSPCEG

LPAADATALT LANRNLERLP GCLPRTLRSL DASHNLLRAL STSELGHLEQ

LQVLTLRHNR IAALRWGPGG PAGLHTLDLS YNQLAALLPC TGPALSSLRA

LALAGNPLRA LQPRAFACFP ALQLLNLSCT ALGRGAQGGI AEAAFAGEDG

APLVTLEVLD LSGTFLERVE SGWIRDLPKL TSLYLRKMPR LT**TLEGDIFK**

MTPNLQQLDC QDSPALASVA THIFQDTPHL QVLLFQNCNL SSFPPWTLDS

18mer

SQVLSINLFG NPLTCSCDLS WLLTDAKRTV LSRAADTMCA PAAGSSGPFS

ASLSLSQLPG VCQSDQSTTL GASHPPCFNR STYAQGTTVA PSAAPATRPA

GDQQSVSKAP NVGSRTIA**AW** **PHSDAREGTA** **PSTTNS**VAGH SNSSVFPRAA

C+18mer

STTRTQHRGE HAPELVLEPD ISAASTPLAS KLLGPFPTSW DRSISSPQPG

QRTHATPQAP NPSLSEGEIP VLLLDDYSEE EEGRKEEVGT PHQDVPCDYH

PCKHLQTPCA ELQRRWRCRC PGLSGEDTIP DPPRLQGVTE TTDTSALVHW

CAPNSVVHGY QIRYSAEGWA GNQSVVGVIY ATARQHPLYG LSPGTTYRVC

VLAANRAGLS QPRSSGWRSP CAAFTTKPSF ALLLSGLCAA SGLLLASTVV

LSACLCRRGQ TLGLQRCDTH L**VAYKNPAFD** **DYPLGLQTVS**

C+19mer

＊ **C20orf75-A：TLEGDIFKMTPNLQQLDC (18mer)**

＊ **C20orf75-B：C＋AWPHSDAREGTAPSTTNS (19mer)**

＊ **C20orf75-C：C＋VAYKNPAFDDYPLGLQTVS (20mer)**

Supplementary Figure 2 (continued)

1.UPK3B isofrom a (NM_030570).gnu
2.UPK3B isofrom b (NM_182684).gnu
3.UPK3B isofrom c (NM_182683).gnu

```
1:MGLPWGQPHLGLQMLLLALNCLRPSLSLGEWGSWMDASSQTQGAGGPAGVIGPWAPAPLR 60

1:-------------------------------------------------------MGLPW 5
1:-------------------------------------------------------MGLPW 5
                                                         .....


61:LGEAAPGTPTPVSVAHLLSPVATELVPYTPQITAWDLEGKVTATTFSLEQPRCVFDGLAS 120
6 :GQPHLGLQMLLLALNCLRPSLSLELVPYTPQITAWDLEGKVTATTFSLEQPRCVFDGLAS 65
6 :GQPHLGLQMLLLALNCLRPSLSLELVPYTPQITAWDLEGKVTATTFSLEQPRCVFDGLAS 65
   ................*......*************************************


121:ASDTVWLVVAFSNASRGFQNPETLADIPASPQLLTDGHYMTLPLSPDQLPCGDPMAGSGG 180
66 :ASDTVWLVVAFSNASRGFQNPETLADIPASPQLLTDGHYMTLPLSPDQLPCGDPMAGSGG 125
66 :ASDTVWLVVAFSNASRGFQNPETLADIPASPQLLTDGHYMTLPLSPDQLPCGDPMAGSGG 125
    ************************************************************
             C+16mer                                      20mer

181:APVLRVGHDHGCHQQPFCNAPLPGPGPYREDPRIHRHLARAAKWQHDRHYLHPLFSGRPP 240
126:APVLRVGHDHGCHQQPFCNAPLPGPGPYRVKFLLMDTRGSPRAETKWSDPITLHQGKTPG 185
126:APVLRVGHDHGCHQQPFCNAPLPGPGPYREDPRIHRHLARAAKWQHDRHYLHPLFSGRPP 185
    *****************************.............................*.
                                 C+18mer

241:TLGLLGSLYHALLQPVVAGGGPGAAADRLLHGQALHDPPHPTQRGRHTAGGLQAWPGPPP 300
186:SIDTWPGRRSGSMIVITSILSSLAGLLLAFLAASTMRFSSLWWPEEAPEQLRIGSFMGK 245
186:TLGLLGSLYHALLQPVVAGGGPGAAADRLLHGQALHDPPHPTQRGRHTAGGLQAWPGPPP 245
    .......................*....*....*.................*........
                                                 C+18mer

301:QPQPLAWPLCMGLGEMGRRE-----------                         320
246:RYMTHHIPPSEAATLPVGCKPGLDPLPSLSP                         276
246:QPQPLAWPLCMGLGEMGRRE-----------                         265
    .......*............
```

＊ **UPK3B-A：C＋SNASRGFQNPETLADI (17mer)**

＊ **UPK3B-B：CGDPMAGSGGAPVLRVGHDH (20mer)**

＊ **UPK3Bb-A：C＋KFLLMDTRGSPRAETKWS (19mer)**

＊ **UPK3Bb-B：C＋EAPEQLRIGSFMGKRYMT (19mer)**
